# A Critical Overview of HPLC-MS-Based Lipidomics in Determining Triacylglycerol and Phospholipid in Foods

**DOI:** 10.3390/foods12173177

**Published:** 2023-08-23

**Authors:** JuDong Yeo, JaeYoon Kang, HyeonJin Kim, Chaeeun Moon

**Affiliations:** Department of Food Science and Biotechnology of Animal Resources, Konkuk University, Seoul 05029, Republic of Korea; copp102@konkuk.ac.kr (J.K.); aibxo@konkuk.ac.kr (H.K.); m00nfull@konkuk.ac.kr (C.M.)

**Keywords:** HPLC-MS/MS, lipid extraction, informatics, food processing, food adulteration

## Abstract

With the current advancement in mass spectrometry (MS)-based lipidomics, the knowledge of lipidomes and their diverse roles has greatly increased, enabling a deeper understanding of the action of bioactive lipid molecules in plant- and animal-based foods. This review provides in-depth information on the practical use of MS techniques in lipidomics, including lipid extraction, adduct formation, MS analysis, data processing, statistical analysis, and bioinformatics. Moreover, this contribution demonstrates the effectiveness of MS-based lipidomics for identifying and quantifying diverse lipid species, especially triacylglycerols and phospholipids, in foods. Further, it summarizes the wide applications of MS-based lipidomics in food science, such as for assessing food processing methods, detecting food adulteration, and measuring lipid oxidation in foods. Thus, MS-based lipidomics may be a useful method for identifying the action of individual lipid species in foods.

## 1. Introduction

Lipids are essential components of cell membranes and are vital for cellular metabolism and the regulation of various cellular and physiological responses, where they function as hormones, energy sources, and signaling molecules [1]. Many plant- and animal-based foods contain a wide range of lipid species with different chemical structures attributed to discrepancies in factors such as the number of double bonds and length and the regiospecificity of acyl chains [2]. Lipid species can be classified into eight major groups based on the type of head group and linkage between aliphatic acyl chains and the head group: fatty acyls, sphingolipids, glycerolipids, glycerophospholipids, prenol lipids, sterol lipids, saccharolipids, and polyketides [3,4]. Most of these lipid species have lower molecular weights (<2000 Da) than carbohydrates and proteins.

Lipids (edible oils and fats) play crucial roles in human health by providing essential nutrients, cellular regulators, and metabolic energy. Recently, patterns in dietary intake and quality of lipids have significantly altered [5], and this led to an increase in the incidence of lipid-related chronic diseases, including type 2 diabetes, obesity, hypertension, cardiovascular diseases, and liver diseases [6], which are currently considered the leading causes of death [7]. Much evidence demonstrated that particular lipid compounds (i.e., conjugated linoleic acids and their isomers, prostaglandin E2) play a significant role in diminishing the risk of non-communicable diseases such as cancer, CVD, and wound healing [8,9,10,11].

**Figure 1:**
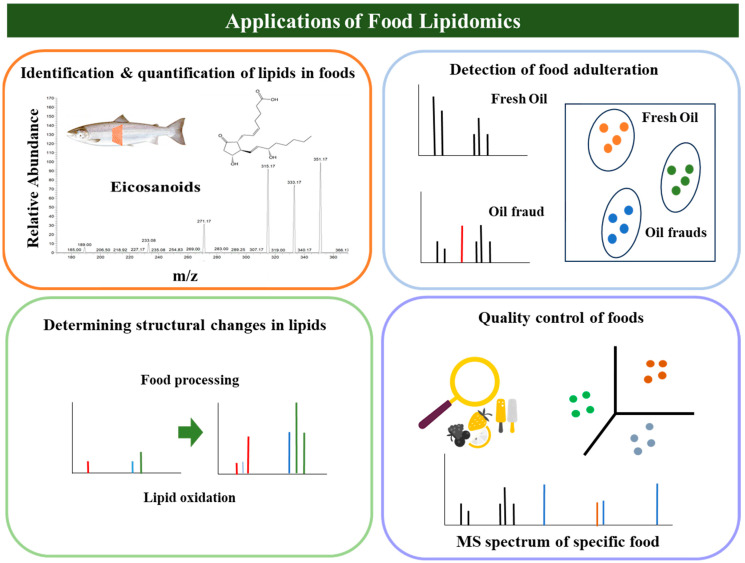
Applications of mass spectrometry-based lipidomics in food science.

Lipidomics is a valuable and effective method for exploring the properties of lipids and their physiological functions, as well as for conducting large-scale and comprehensive studies such as food science, biology, biochemistry, and clinical studies (i.e., disease diagnosis, clinical biomarker discovery, and in understanding disease pathology) [1]. Mass spectrometry (MS)-based lipidomics, in particular, can monitor the change in individual lipid species in foods under certain environments, such as food processing [12]. Several factors interfere with the accurate and precise identification and quantification of lipid species in foods, owing to the presence of various lipid isomers and adduct formations [13]. Rapid advances in MS, particularly soft ionization and tandem mass spectrometry (MS/MS), have led to the identification of more than 100 lipid molecules with a single injection [12]. This high-throughput approach provides useful information on the structural properties of the identified lipids [14]. Moreover, lipidomics is a valuable tool in detecting food adulteration, monitoring quality control, and screening the structural alteration of lipids in foods upon processing and oxidation (Figure 1) [15,16].

Lipidomics has been widely applied to identify and quantify lipid species in various foods. However, the workflow and wide applications of MS-based lipidomics have been poorly demonstrated in the food science field. Thus, the present work aims to offer an in-depth review of MS-based lipidomics and their effectiveness in characterizing lipid molecules in plant- and animal-based foods. In addition, this study provides information on the usefulness of lipidomics analysis for assessing food processing methods, detecting food adulteration, and measuring lipid oxidation.

## 2. Functions of Lipid Species

Lipids play an important role in food. It serves as a source of energy, imparts texture and develops order and flavor, and provides various properties to foods. Lipids in fats and oils also play a role in creating the unique texture of food. It also acts as an emulsifier in forming various types of emulsion systems.

In plant- and animal-based foods, numerous lipid species coexist with other molecules, such as proteins and carbohydrates, in the cellular space, where complex metabolic processes occur through diverse pathways and networks; thus, lipid species play significant roles in sustaining healthy cell life [17]. The primary function of lipid species in the cell is to serve as the main building blocks in the construction of the lipid membrane. Diverse subclasses of phospholipids, including phosphatidylcholine (PC), phosphatidylethanolamine (PE), phosphatidylinositol (PI), phosphatidylglycerol (PG), and phosphatidylserine (PS), are involved in lipid bilayer formation [17]. Sphingomyelin (SM), cardiolipin, cholesterol and its esters (e.g., galactosylceramide), and other molecules are also important materials required for lipid membrane formation.

The lipid membrane acts as a permeable barrier for cells and organelles, and its physical characteristics play a significant role in cell functions [18]. Moreover, lipid species are stored as energy sources and are used for cell metabolism. Lipid molecules, including triacylglycerol (TAG), diacylglycerol (DAG), and monoacylglycerol (MAG), are stored as lipid droplets and localized in adipose tissue. Further, acyl-CoA and acylcarnitine are involved in cellular metabolism. Lipids such as fatty acids are broken down into acyl-CoA, which is then used in the production of adenosine triphosphate upon entering the citric acid cycle. Acylcarnitines are biosynthesized from carnitine and acyl-CoAs by carnitine acyltransferases in peroxisomes or mitochondria [19]. They are also used for energy generation in mitochondria and the production of other molecules [20]. Another significant function of lipid molecules is their signaling behavior; for example, all lysolipids, DAG, MAG, acyl-CoA, acylcarnitine, ceramide, sphingosine, sphingosine-1-phosphate, psychosine, steroids, and N-acylethanolamine are representative signaling molecules [17].

## 3. Lipidomics

Many lipid molecules have been identified from plant- and animal-based foods, as well as from microorganisms. Lipidomics, which is assigned in metabolomics, is a useful method for understanding the interactions between lipids and neighboring molecules [21]. Moreover, it is a practical analytical chemistry technique to study the metabolism of cellular lipids [17]. Remarkable improvements in MS have enabled the efficient identification and quantification of lipid molecules in various plant- and animal-based foods. Moreover, MS-based lipidomics provides useful information on the structure of lipid molecules through the fragmentation of target ions [22]. Several MS-based imaging (MSI) techniques have been developed by coupling MS with desorption electrospray ionization (DESI), matrix-assisted laser desorption/ionization (MALDI), and/or time-of-flight (TOF) secondary ion, which have proven to be suitable tools for exploring the spatial distribution of lipid species in tissues and cellular spaces without an extraction process [23,24,25]. Detailed information on the principles and applications of MS-based imaging techniques is discussed later. Furthermore, the application of advanced MS techniques, such as quadrupole TOF MS and quadrupole Orbitrap Fourier transform MS, have facilitated in-depth analysis and precise detection of diverse lipid species [26]. In food science, lipidomics has been utilized for identifying and quantifying lipid species from various food sources, as well as to understand the alterations in their structure caused by food processing, assess food safety, and evaluate food quality assurance [1].

Untargeted lipidomics is a broad-scale technique that covers a wide range of target compounds from polar lipids (i.e., lysophospholipids, phospholipids, monoacylglycerol) to very hydrophobic species such as triacylglycerols [27,28]. There is no single platform to detect the entire lipid molecules: developing methods for untargeted analyses mainly focus on encompassing the broad range of lipidomes while sustaining high accuracy and precision for the lipid classes detected by the employed platforms. On the other hand, classical targeted lipidomics strategies aim for higher accuracy and precision of analysis for the narrow range of desired lipid molecules, along with optimized analytical parameters for individual species. Thus, one should choose proper approaches, untargeted or targeted lipidomics, based on their primary focus.

## 4. Lipidomics Workflow

**Figure 2:**
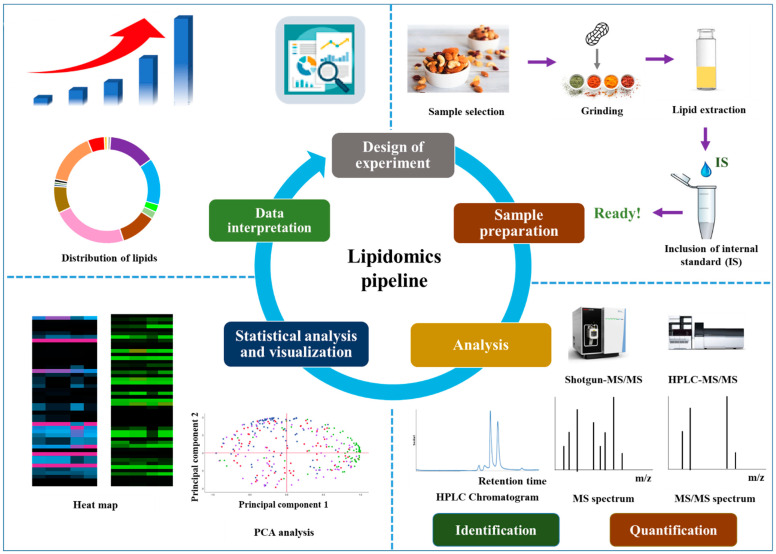
Mass spectrometry-based lipidomics pipeline in analyzing food-derived lipids demonstrating the steps from sample preparation to data interpretation. Different colors represent different groups of samples.

The lipidomics workflow includes complex steps and processes such as lipid extraction, induction of adduct formation, MS analysis, data processing, statistical analysis, and informatics to obtain the desired information (Figure 2). This section describes the basic principles of each lipidomics step and the practical information required for the successful implementation of food lipidomics.

### 4.1. Induction of Adduct Formation

Prior to injection into the mass spectrometer, adduct formation is generally induced by the addition of appropriate salts to the samples/lipid extracts in shotgun MS or to the mobile phase in the high-performance liquid chromatography (HPLC)-MS/MS system to strengthen the ionization of the molecules to be analyzed. This approach is particularly useful for analyzing molecules that are not readily ionized by MS [29,30]. A suitable salt can effectively enhance the sensitivity in detecting target molecules by up to three orders of magnitude [31].

Adduct formation has been extensively applied in lipidomics analysis, particularly the formation of sodium ([M + Na]^+^), ammonium ([M + NH_4_]^+^), and potassium ([M + K]^+^) adducts [31]. Yeo and Parrish [32] analyzed the sodium adducts of TAG species extracted from salmon muscle tissue using shotgun electrospray ionization (ESI)-MS, which demonstrated the effectiveness of generating sodium adducts of TAG molecules for their profiling and quantitative analysis. Koivusalo et al. [33] reported that phospholipids exhibit a discrepancy in instrumental response depending on the type of adduct formed (protonated, sodium, or ammonium adduct), indicating the significance of inducing the appropriate adduct formation in MS to obtain the desired information.

### 4.2. Mass Spectrometry Analysis

#### 4.2.1. HPLC-MS and Shotgun MS

HPLC- and shotgun MS analyses are the two main methods for injecting samples into a mass spectrometer. This section provides detailed information on the basic principles and practical uses of HPLC- and shotgun MS. MS is typically combined with HPLC, which is a continuous-flow analytical system that enables the separation of individual molecules in a sample based on their polarities [34]. After the separation in the HPLC system, these molecules are introduced into the mass spectrometer system through diverse interfaces.

Unlike the HPLC-MS/MS system, shotgun MS analysis directly injects the prepared samples into the mass spectrometer without separating the molecules. The primary advantage of shotgun MS is the unlimited infusion of a sample into the mass spectrometer at a constant concentration [17]. This enables immediate adjustment of MS analysis conditions, including polarity (i.e., positive or negative mode), gas flow, collision-induced dissociation (CID) energy, and scanning modes (such as product ion, precursor ion, and neutral loss scanning). This flexibility of shotgun MS analysis facilitates efficient identification of the target molecules in the samples by readily adjusting or optimizing the mass spectrometer analysis conditions.

Meanwhile, GC-MS-based lipidomics is a valuable approach for analyzing lipid species with relatively lower molecular weight, such as free fatty acids (FFAs), steroids, and their esters, after an adequate derivatization process [35]. The most common derivatization reagent used for silylation is N-methyl-N-(trimethylsilyl)trifluoroacetamide (MSTFA) and ammonium iodide (NH_4_I). Here, trimethylchlorosilane (TMCS), dithioerythritol (DTE), trimethylsilylimidazole, or trimethyliodosilane (TMSIm) are often utilized to accelerate the above reaction [36]. Fatty acid methyl ester (FAME) derivatization by inducing methylation of fatty acids is also widely used in global fatty acid profiling of food products.

#### 4.2.2. Scanning Modes in MS Analysis

The ionized molecules introduced into the analyzer first pass through the MS1 compartment responsible for selecting the target parent ions or scanning all molecules in the samples. The parent ions are then transferred into the CID system, referred to as the collision cell, for the fragmentation process, which yields the product ions. The generated product ions finally reach the second analyzer (MS2), where they are filtered depending on the scanning mode.

MS/MS analysis involves four main scanning modes: product-ion scanning, selected reaction monitoring (SRM), neutral-loss scanning, and precursor-ion scanning. In product-ion scanning mode, the parent ions in MS1 enter the CID system and undergo fragmentation under certain dissociation conditions, generating product ions. The product ions are then scanned in MS2 and subsequently expressed in the MS spectrum, as shown in the figure.

SRM is a targeted scanning approach suitable for identifying target molecules, particularly relatively small molecules (such as metabolites and drugs), with known fragmentation patterns under specific MS conditions. Recently, the application of SRM has been extended to larger molecules, such as proteins, indicating its usefulness and wide application range [37]. In SRM, a specific molecule is selected in MS1 by filtering undesired molecules. The selected parent ions undergo fragmentation in the collision cell, generating product ions, followed by the selection of a specific product ion in MS2.

Unlike the product-ion scanning and SRM modes, the neutral-loss scanning mode has a different process: all parent ions are scanned and recorded in the MS1 stage. Subsequently, all the parent ions introduced into the CID undergo fragmentation. Specific product ions that show the desired neutral loss are transferred to the MS2. Later, the product ions are scanned, and the MS system tracks parent ions possessing such product ions, leading to the expression of the intensities of the parent ions in the MS spectrum.

The principle of the precursor-ion scanning mode is similar to that of the neutral-loss scanning mode in the MS1 and CID stages; however, the main difference between the two modes lies in the MS2 stage. Here, the MS2 stage selects specific product ions designated by the operator, leading to the display of the parent ions containing the detected product ions.

In mass spectrometry-based lipidomics, the different analysis conditions (i.e., interfaces, ionization sources, and scanning modes) are used depending on sample origins and target compounds. For instance, Zhang et al. [38] utilized a 3000 UHPLC (Dionex) connected with a QExactive MS system along with ESI and product ion scan mode to determine the TAG profiles of several fishes such as big eye tuna, bighead carp, and Atlantic salmon. In addition, multiple neutral-loss scans were employed to identify and quantify individual TAG species in salmon muscle tissue in the positive mode [32]. Gang et al. [39] utilized shotgun mass spectrometry with precursor-ion and neutral loss scanning to determine the composition of phospholipids in edible whelks. The above examples show that versatile MS conditions and strategies can be used in lipidomics analysis depending on the desired results.

### 4.3. Bioinformatics and Statistical Analysis

After MS analysis, a large amount of raw data (information) is obtained from the lipidomics analysis, with the type of data varying depending on the analysis strategies and instruments used. Bioinformatics analysis has become an essential technique for identifying and quantifying targeted or untargeted molecules from generated raw data. Moreover, it can elucidate the structures of lipid species and their metabolic pathways [40]. Various online tools are available for the identification, classification, and prediction of lipid molecules and their structures based on the acquired MS spectra [41]. For instance, LipidBank (http://lipidbankjp, accessed on 14 August 2023) is a free database that provides information on various lipid species, such as fatty acids, glycerolipids, steroids, sphingolipids, and vitamins. Lipid maps (http://www.lipidmaps.org, accessed on 14 August 2023), another online resource, has been utilized for the classification of a wide range of lipid species [1]. Cyberlipid Center (http://www.cyberlipid.org, accessed on 14 August 2023) provides detailed descriptions and structural information on individual lipid molecules. Some tools such as SphinGOMAP (http://sphingolab.biology.gatech.edu, accessed on 14 August 2023) and Kyoto Encyclopedia of Genes and Genomes (KEGG) (http://www.genome.jp/kegg, accessed on 14 August 2023) are useful for studying bioreactions (metabolic pathways and pathway maps for biosynthesis) of lipid molecules. Further, several types of software have been widely used for data processing, with each software having different data processing functions such as peak detection, normalization, standardization, annotation, isotopic deconvolution, data visualization, and multivariate statistical analysis of the data [42]. Other practical software used in lipidomics includes MZmine 2 (http://mzmine.sourceforge.net, accessed on 14 August 2023) and LipidX (http://www.systemsx.ch, accessed on 14 August 2023). As each tool provides different information, selecting an appropriate program is essential for obtaining the desired results. Meanwhile, the sharing of mass spectrometric information such as spectra, fragment ions, and analysis conditions among relevant researchers enhances the development of MS-based lipidomics and broadens its applications. Moreover, LIPID MAPS Structure Database (LMSD) encompasses more than 47,000 lipids acquired from other sources such as lipid databases, experimental work carried out by the LIPID MAPS consortium, computationally generated on the basis, and the scientific literature [43]. LMSD enables either bulk annotations for MS data based on the shorthand nomenclature, as described by Liebisch et al. [44], or fully annotated names in case users already possess supplementary structural information by MS/MS experiments.

Chemometrics is a data analysis technique used to recognize patterns and cluster samples into groups based on their similarity, as well as to identify the main molecular species that represents a specific phenotype [45]. It improves the quality of datasets after identification and quantitative analysis of the lipid species using the aforementioned software. Subsequently, various classical methods, including principal component analysis (PCA), analysis of variance, and partial least squares-discriminant analysis (PLS-DA), can be used to organize complex datasets [46]. Additionally, two-dimensional chromatography combined with MS and multivariate curve resolution, a method that allows background effects to be controlled in two different ways based on analytical conditions, has also been utilized to create useful diagrams that facilitate the organization of complicated datasets [46,47]. Bioinformatics techniques, such as KEGG, also enable the interpretation of alterations in lipid molecules during biological or chemical reactions by suggesting possible reaction mechanisms using pathway analyses [48]. Moreover, the regions of interest multivariate curve resolution (ROIMCR) technique can filter complex datasets and organize the regions of the target molecules in the m/z domain. It enables the organization of massive datasets by filtering them based on the ROI without decreasing spectral accuracy [49]. The experimental strategies used to obtain MS data influence the subsequent data processing and informatics analysis; thus, different bioinformatics techniques can be employed for the efficient analysis of shotgun- and HPLC-based MS datasets [50].

## 5. Validation of Lipidomics Analysis

The results acquired from MS analysis and data processing can be validated by alternative approaches; that is, the results from HPLC-MS analysis can be validated using shotgun-based MS analysis and vice versa [17]. Cajka et al. [51] compared lipidomics data from 126 human plasma samples using nine different mass spectrometers: one TOF, one Q/orbital ion trap, and seven Q/TOF. Quantitative analysis showed similar results for MS-based lipidomics in PLS-DA and variable importance in projection scores [51]. Additionally, other methods, such as NMR and chromatography-based analysis, can also be utilized to validate the total lipid content of each lipid subclass.

In addition, bioinformatics analysis of gene and protein expression can be an effective means of validating the data obtained from a lipidomics analysis. In other words, data from transcriptomics and proteomics analyses help in the interpretation of lipidomics analysis. For instance, Momin et al. [52] reported a significant relationship between sphingolipid profiles obtained using HPLC-MS analysis and gene expression data (*p* < 0.001). Moreover, some new sphingolipids suggested by transcriptomics analysis have been confirmed using HPLC-MS; for example, a high level of d16:1 ceramide in cancer cells has been predicted by the elevated expression of relevant enzymes such as serine palmitoyltransferase 3 [53,54]. Thus, combining lipidomics with other effective approaches, such as transcriptomics and proteomics, strengthens its analysis and provides comprehensive insights into the biological reactions of lipid species [17].

## 6. Mass Spectrometry-Based Imaging Techniques

Recently, MSI techniques have been used extensively in a wide range of research fields [48]. For example, Gode and Volmer [55] applied an MSI technique (spatial mapping) to present the lipid species distribution in the cells and tissues of samples after the acquisition of MS data. Such techniques are effective because they display the localization of individual molecules in a sample matrix. Among the ionization approaches, MALDI and DESI have been commonly utilized in MSI techniques because they do not require the complicated lipid extraction steps that are essential for ESI [56]. MALDI-MSI is commonly employed in lipidomics analysis and has been proven to be an effective approach for analyzing phospholipids in animal tissues, including the brain, colon, liver, and heart tissues. It facilitates the tracking of the alterations in phospholipids in cells and tissues, providing useful information on disease states and insights into the prediction of diseases and monitoring of their progression [55,57].

Additionally, MSI techniques have been widely applied in food science because they provide valuable knowledge on the localization of specific molecules in a food matrix, highlighting the desired functional/nutritional compounds for consumption [58]. Various matrices are used for the ionization of molecules in samples, such as 1,5-diaminonapthalene, 2-mercaptobenzothiazole, dihydroxyacetone phosphate, 2,5-dihydroxybenzoic acid, and 9-aminoacridine [58]. Several studies have investigated the localization of lipid species in foods using MSI. For instance, Goto-Inoue et al. [59] investigated the different localizations of TAG molecules in wild and farmed red sea bream using MSI and found that TAG molecules (e.g., 16:1_22:6_22:6) are localized in the muscle tissue of wild and farmed red sea bream. Enomoto et al. [60] studied the distribution of PC species in different pork tissues, including spinalis muscles, transparent tissues, and intermuscular fat, using MALDI-MSI. The results showed that alkylacyl-, alkenylacyl-, and diacyl-PC molecules exhibited unique distribution patterns in the analyzed tissues, indicating that the distribution of PC molecules is significantly affected by the composition of the fatty acyl chains in their structure. Enomoto et al. [61] also utilized MALDI-MSI to explore the distribution of SM species in pork chops, such as intermuscular fat tissue, loin, spinalis muscle, and transparent tissues. The results demonstrate that SM molecules possessing stearic acid are mainly localized in the spinalis muscle and loin, whereas SM species containing palmitic, nervonic, and lignoceric acids are predominantly localized in transparent tissues, proving the usefulness of the MSI technique in visualizing the distribution of SM species in animal-based foods.

In summary, extensive developments in MSI techniques have improved our understanding of the distribution of specific lipid species in plant- and animal-based foods. Thus, this approach is helpful for investigating the localization of individual lipid species in foods.

## 7. Lipidomics in Plant-Based Foods

### 7.1. TAGs

TAGs are major energy storage molecules in plants and are widely used as food, feed, and feedstock for biofuel production [62]. In addition, TAG metabolism is involved in cell division, membrane lipid remodeling, stomatal opening, organ formation, and pollination in vegetative tissues [63]. Recently, increasing evidence has demonstrated the successful application of MS-based lipidomics in food science. In this section, a comprehensive overview of the practical use of MS-based lipidomics for identifying and quantifying TAGs in foods is provided with various examples.

The wide applications of MS-based lipidomics in the determination of TAG profiles in plant-based foods are summarized in Table 1. Further, MS-based lipidomics has been used as an effective approach for detecting adulteration and authentication of soy milk by TAG profiling and subsequent quantitative analysis. Li et al. [64] identified 14 lipid molecules using this approach, which serve as markers for detecting milk fraud. Li et al. [65] employed multiple neutral scanning modes to identify and quantify TAG molecules in soybean oil using a shotgun lipidomics approach. They identified 93 TAG molecules and the major TAG molecules in soybean oils, which included 18:2_18:2_18:2 (LLL), 18:2_18:2_18:3 (LLLn), 18:1_18:2_18:2 (OLL), 16:0_18:2_18:2 (OLLn), 16:0_18:1_18:2 (POL), 18:1_18:1_18:2 (OOL), and 18:0_18:2_18:2 (SLL). Lísa and Holčapek [66] used a chiral HPLC-APCI-MS method coupled with two cellulose tris-(3,5-dimethylphenylcarbamate) columns in a series to investigate the TAG regioisomers and enantiomers in hazelnut oil. They found that unsaturated fatty acids are highly localized in the sn-2 position in hazelnut oil, demonstrating that MS-based lipidomics is an efficient method for determining TAG profiles and their regioisomers in foods. Dong et al. [67] employed an APCI-MS technique to determine the primary discrepancy in TAG species (i.e., 18:1_18:1_18:1, 16:0_18:1_18:2, and 18:1_16:0_18:1) between high-oleic and normal peanut oils by creating a multidimensional data matrix using PCA. Moreover, MS-based lipidomics is a useful tool for observing acyl migration and monitoring specific TAG molecules in reactions. For instance, Liu et al. [68] used the HPLC-APCI-MS/MS approach to monitor the levels of 1,3-dioleoyl-2-palmitoylglycerol, a significant TAG molecule in human milk, in the lipase-catalyzed synthesis of structured lipids.

Therefore, MS-based lipidomics has been proven to be a valuable approach for determining TAG profiles in plant-based foods and their quantitative analysis, as well as for characterizing specific TAG molecules, detecting food adulteration, and monitoring structural alterations of TAG molecules.

### 7.2. PLs

MS-based lipidomics has also been used successfully to identify and quantify phospholipid profiles. For instance, Capriotti et al. [69] used an exactive hybrid quadrupole–Orbitrap mass spectrometer to identify 19 phospholipids in extra-virgin olive oil. Moreover, they monitored the degradation of these phospholipids during storage and concluded that the lipidomics approach is useful for studying the mechanisms of hydrolysis and oxidation of olive oil. Shen et al. [70] used MALDI-TOF-MS to screen more than 60 phospholipid molecules in almonds and proposed that the ratio of m/z 833.6 to 835.6 and m/z 821.6 could be an efficient marker for distinguishing almonds with different geographical origins.

The use of MS-based lipidomics in the evaluation of PL profiles in plant-based foods is provided in Table 1. Furthermore, Alves et al. [71] used MS-based lipidomics to identify polar lipids in olive fruits (*Olea europaea* L.); 107 polar lipids belonging to 11 lipid subclasses, including phospholipids, glyceroglycolipids, glycosphingolipids, and betaine lipids, were identified in *O. europaea* fruits. Anagbogu et al. [72] used ultra-performance liquid chromatography coupled with MS (UPLC-MS) to screen lipid molecules in 30 genotypes of *Coffea canephora* L. beans cultivated in Southwestern Nigeria. They found that PE (34:2) is the predominant phospholipid species in *C. canephora* beans. Yang et al. [73] used lipidomics to screen PCs in six types of beans using UHPLC-Q-Exactive Orbitrap-MS and identified 49 PC species, among which 18:2_18:2_PC was the predominant PC molecule in soybean, red kidney bean, red bean, and white kidney bean. Moreover, the results demonstrated that 16:0_18:1_PC was high in chickpea PC, and diverse plasmanyl PC species and docosahexaenoic acid (DHA)-containing PC species were detected in the six types of beans. MS-based lipidomics has also been used to monitor the alteration of lipid species at several different developmental stages of the model plant *Arabidopsis thaliana*, and more than 200 lipid species from different subclasses have been identified from each developmental stage [74]. In summary, MS-based lipidomics has proven to be a useful technique for determining lipid profiles and has shown excellent performance in monitoring the alteration of lipid species in processed foods or foods affected by environmental factors.

## 8. Lipidomics in Animal-Based Foods

### 8.1. TAGs

Many cells and organs of eukaryotes and some prokaryotes can synthesize TAG molecules, with organs such as the liver, intestine, and adipose tissue having a more dynamic capacity than others. In general, eukaryotic organisms synthesize TAG molecules and store them as lipid droplets enclosed by a monolayer of phospholipids in adipose cells/tissue, which are then used during fasting conditions or high-energy demand situations [75].

Lipidomics analysis for TAG analysis in animal-based foods is summarized below. Lipids in animal-based foods primarily comprise TAGs; phospholipids; and other minor components, including free fatty acids (FFAs), MAGs, DAGs, wax esters, sterols, and lipid-soluble vitamins [1]. Numerous studies have reported the use of MS-based lipidomics for the identification and quantification of individual TAGs in animal-based foods. Rocchetti et al. [76] employed MS-based lipidomics to monitor the alterations in lipid species in pigs fed two different diet supplements, n-3 fatty acids and polyphenols obtained from grape skin and oregano. They detected 1507 lipid molecules and 195 compounds matching the MS/MS spectra provided by the LipidBlast database, in which they found alterations in 32 TAG species when the pigs were fed two different diets. Moreover, they attempted to combine lipidomics and transcriptomics and concluded that an n-3 fatty acid diet prevents adipogenesis and inflammation processes. Robson et al. [77] demonstrated that untargeted lipidomics using rapid evaporative ionization MS (REIMS)-based analysis exhibited excellent performance in detecting beef fraud.

Lipidomics has also been used to identify lipid species in marine sources. For instance, Yeo and Parrish [32] applied shotgun-based lipidomics to identify individual TAG species in salmon muscle tissue and for their quantitative analysis, which revealed 98 TAG molecules in total by employing multiple neutral-loss scans. Salmon muscle tissue was found to contain a wide range of TAG species with diverse fatty acyl chain combinations, among which 18:0_18:1_22:6 (16.4%), 16:0_18:0_20:5 (10.4%), and 18:1_18:2_22:6 (9.0%) were the main TAG species, demonstrating that a high proportion of TAG molecules possess ω-3 fatty acids such as DHA and eicosapentaenoic acid (EPA). Zhang et al. [38] utilized a UHPLC-Q-Exactive-MS system to determine the TAG profiles in bigeye tuna, bighead carp, and Atlantic salmon heads, leading to the identification of 146, 87, and 90 TAG molecules, respectively. Donato et al. [78] employed MS coupled with multiple HPLC systems to identify and quantify TAG molecules in the Mediterranean mussel *Mytilus galloprovincialis* and found 34 TAG molecules using the product ion scanning mode. Moreover, Zhang et al. [79] identified and quantified 23 TAG molecules in anchovy and tuna oils and demonstrated that DHA- and EPA-containing TAGs, such as 16:0_18:1_20:5, 18:1_22:6_22:6, and 16:0_18:0_22:6, were the major TAG molecules in these oils using HPLC-APCI-MS.

Thus, MS-based lipidomics has been widely employed for the determination of TAG profiles and their quantitative analysis in various animal-based foods. Different types of advanced mass spectrometers and diverse scanning modes have been utilized to facilitate effective lipidomics studies, and it has been proven that MS-based lipidomics is an excellent approach for characterizing lipid species in a wide range of animal-based foods as well as for understanding the distribution of specific fatty acids such as DHA and EPA in TAG molecules and their proportions.

### 8.2. PLs

In general, 60 mol% of the lipids in eukaryotic cells are phospholipids, which play a crucial role in sustaining cellular life [80]. Phospholipids have amphiphilic properties because they possess both hydrophilic (polar head) and hydrophobic (nonpolar tail) portions and are the main building blocks of the cell membrane, as they form a lipid bilayer. PC and PE are the primary phospholipid molecules that form the lipid membrane. The outer membrane mainly consists of PC molecules, whereas the inner membrane is mainly composed of PE and PS molecules [81].

Moreover, phospholipids, particularly those containing ω-3 fatty acids (such as DHA and EPA) present in marine sources, have a variety of health benefits, including inhibiting hypercholesterolemia, neurological disorders, and liver ailments and controlling the immune system by activating specific or nonspecific defense systems [82,83].

MS-based lipidomics has been applied to a wide range of animal-based food samples. Detailed information on the lipidomics analysis conducted by various studies and their findings are summarized in Table 2. Shen et al. [84] compared the efficiency of classical and modified lipid extraction methods by performing MS-based lipidomics with both gas chromatography (GC)–flame ionization detection (FID) and hydrophilic interaction liquid chromatography (HILIC)–QTrap to compare differences in lipid profiles depending on the lipid extraction method used. They used different methods to isolate lipid molecules from shrimp waste and found that the extraction methods provided similar lipid profiles and levels of each molecular species. Yeo and Parrish [2] investigated the phospholipid profiles in salmon muscle tissue using shotgun MS and identified 43 phospholipid species belonging to four different classes: PCs, PEs, PSs, and PIs, with the predominant phospholipid molecule being 16:0–22:6 PtdCho [M + Na]^+^ at m/z 828.4, accounting for more than 50% of the PC species. They also found that most phospholipids contained ω3 polyunsaturated fatty acids (ω3 PUFAs), such as DHA and EPA. MS-based lipidomics has also been employed to determine the phospholipid profile in Atlantic salmon, king salmon, and rainbow trout; 37 phospholipid species were identified and quantified, and 18:0_20:5_PE, 18:1_20:5_PE, 18:0_20:5_PE, 18:0_22:6_PE, and 18:0_22:6_PI were the predominant phospholipid species in the three fish species [85]. Boselli et al. [86] applied lipidomics to determine the phospholipid molecules present in sea fish, freshwater fish, and shellfish using HPLC-MS/MS and found 18 PCs, 24 PEs, 15 PSs, and 8 PIs in these species, with several of these phospholipids containing ω3 PUFAs in their structure. Further, Zhang et al. [87] used HILIC-MS to understand the phospholipid profiles in the crab *Portunus trituberculatus*. They identified several DHA- and EPA-containing phospholipids, such as 18:0_20:5_PC, 18:1_20:5_PE, 16:0_22:6_PE, and 18:0_20:5_PI, suggesting that MS-based lipidomics could be a high-throughput tool for monitoring polyunsaturated phospholipid molecules in foods. Moreover, MS-based lipidomics with UPLC-Q-Exactive Orbitrap/MS has been used to determine phospholipid profiles in shrimp heads, codfish roes, and squid gonads, in which 310 phospholipid molecules with 34 different fatty acyl chain combinations were simultaneously identified using the LipidSearch software [88].

MS-based lipidomics has also demonstrated a rapid and high-throughput performance in determining lipid species of other sample origins. For instance, Jia et al. [89] studied the alteration of phospholipid species in Tan sheep meat treated by thermal processing using UHPLC-Q-Orbitrap-based lipidomics. They identified and quantified 90 lipid species belonging to six lipid subclasses, with a focus on phospholipids. Li et al. [90] used an extraction medium containing a mixture of methanol and chloroform (2:1) to isolate phospholipids from duck meat and used shotgun lipidomics to monitor the alteration in the levels of individual phospholipids during water-boiled salted duck processing. Significant alterations were observed in individual phospholipid molecules during processing. Lipidomics has also been applied to determine phospholipid species in microorganisms. For instance, Couto et al. [91] employed HILIC-MS to study polar lipids in *Chlorella vulgaris* using different extraction methods. More than 30 phospholipids were identified in the lipid extract of *C. vulgaris*, and ultrasonication in lipid extraction increased the yield of polar lipids from C. *vulgaris*.

## 9. Use of Mass Spectrometry-Based Lipidomics in Food Processing

Food processing (e.g., fermentation, germination, roasting, steaming, and boiling) can greatly affect the physical and chemical properties of foods, as well as their composition. Lipid molecules, a major component in foods, also undergo remarkable changes during food processing. With the advancements in diverse MS-based lipidomics techniques, alterations in the lipid species of foods due to various processing have been extensively evaluated by existing studies. Shi et al. [92] screened lipid species in tilapia fillets during steaming, boiling, and roasting via untargeted metabolomics using the UPLC-Q-Exactive Orbitrap MS approach. Napolitano et al. [93] employed LC-ESI/LTQ-Orbitrap-MS/MS to monitor the multi-class polar lipid profiles of fresh and roasted hazelnuts. They identified 120 polar lipid molecules belonging to several subclasses, including phospholipids, sphingolipids, and glycolipids, and measured their alterations upon roasting. Cui et al. [94] investigated the changes in the fatty acid composition of ten types of oils during four frying processes, including stir-frying, vegetable salad, deep-frying, and pan-frying using GC-MS. Trans fatty acid (TFA) was commonly produced during all the frying processes; in particular, rapeseed oil showed the highest TFA level in vegetable salad oil. Moreover, shotgun ESI-tandem MS has enabled rapid lipid profiling of cold-pressed rapeseed oils and their quantitative analysis after microwave processing by developing the method for analyzing TAGs, PLs, and FFAs [95]. GC-MS-based lipidomics has also been employed to screen for changes in the fatty acid profiles of Nigerian African walnut oil during retail processing. Notably, retail processing significantly improves the levels of major fatty acids such as linolenic acid (C18:3, cis-9, 12, 15). Thus, the above evidence clearly shows the usefulness of MS-based lipidomics for identifying and quantifying the lipid composition of foods during different processing methods.

## 10. Application of Lipidomics in Detecting Food Adulteration

Adulteration occurs when a food item does not satisfy the legal standards established by the government; poor-quality substances are added to the food item to expand its volume [96]. In general, there are two types of food adulteration: substitution by poor-grade but similar substances or by exogenous components regardless of food or non-food substances to mask low quality (e.g., adding vegetable oil to milk to enhance the level of fat) [96]. Recently, MS-based lipidomics has been widely used to detect such adulterations by monitoring lipid molecules as markers.

Criado-Navarro et al. [97] determined glycerophospholipid profiles in edible vegetable oils using liquid chromatography coupled with tandem MS (LC-MS/MS) and found that glycerophosphatidic acids and phosphatidylglycerides were the predominant molecules, which were used for discriminating virgin olive oils from other oils. The combination of GC-MS and UPLC-MS has also been applied to analyze metabolites in beef mince adulterated with pork, and pathway analysis has revealed a remarkable discrepancy in the levels of glutathione, sphingolipid, and inositol metabolism [98]. In addition, some lipids that are relatively abundant in certain types of foods (e.g., sterols in milk and lysophospholipids in white rice) have been designated as target molecules for detecting adulteration [99,100]. Creydt and Fischer [101] demonstrated untargeted lipidomics using ion mobility MS to discriminate different species of truffle varieties, including white truffles (*Tuber borchii* and *T. magnatum*) and black truffles (*T. melanosporum*, *T. indicum*, and *T. aestivum*).

## 11. Assessment of Lipid Oxidation Using Lipidomics

Lipid oxidation alters the physical and chemical properties of fats and oils in foods, leading to quality deterioration and resulting in off-flavors, color changes, and alterations in taste and texture [102]. It also produces various oxidation products, including primary (e.g., hydroperoxides and conjugated dienes) and secondary products (e.g., hydrocarbons, alcohols, aldehydes, and ketones). Thus, MS-based lipidomics is an effective method for identifying and quantifying lipid oxidation products.

Wang et al. [103] reported that the drying processes of rape bee pollen cause its oxidation, which is closely related to three main metabolic pathways: glycerophospholipid, glycerolipid, and linoleic acid metabolic pathways. MS-based lipidomics has also been used to determine the oxidation of edible oils. Ito et al. [104] employed a chiral stationary phase LC-MS/MS approach to monitor the oxidation products of linoleic acids in two different oxidation systems: photooxidation and autooxidation. They found that different hydroperoxyoctadecadienoic acid isomers were formed depending on the oxidation system. The LC-MS/MS approach was also used to determine the oxidation of canola oil, in which epoxidized and hydroperoxidized TAG molecules were identified and quantified using LC-MS/MS without additional derivatization or hydrolysis [105].

Lipidomics has also been used to evaluate lipid oxidation in biological samples. For instance, oxygenated lipid products, such as isoprostanes and isofurans, have been utilized as biomarkers of oxidative stress owing to their relationship with certain diseases. Lee and Lee [106] identified and quantified isoprostanes and isofurans in blood and tissue samples using MS. Moreover, Hu et al. [107] employed multidimensional MS-based shotgun lipidomics to profile cellular lipid species; 50 lipid classes and thousands of individual lipid molecules and their alterations upon lipid peroxidation were identified. Spickett and Pitt [56] summarized the effectiveness of MS-based lipidomics in the investigation of oxidized phospholipids in biological systems with a focus on oxidized phospholipids in animal models and clinical samples, along with the recent improvements in MS, particularly the use of the fragmentation pattern of the target molecular ion and enhanced resolution and accuracy. Thus, MS-based lipidomics has proven to be a useful tool for studying lipid oxidation in foods as well as biological systems.

## 12. Recent Important Advances in MS-Based Lipidomics

There were many advances in MS-based lipidomics in exploring lipid isomer characterization; for instance, Zhao et al. [108] developed a workflow for determining PC profiles with *sn-* and double-bond positions at high sensitivity. Multi-dimension techniques have also been developed for wide-range lipid analysis. In two-dimensional (2D) separations, the effluents from the first dimension are transferred to a rapid 2D separation online, or fractions are collected offline and independently analyzed on the 2D. [109,110]. Further, advances in MS methods for the point-of-care analysis of lipid biomarkers allow us to obtain effective clinical results when the patients are on-site [111]; however, developing effective technologies that can address the high cost of MS use remains a challenge.

## 13. Conclusions

This review summarizes the principles of MS-based lipidomics for different foods and the detailed procedures used for lipid extraction, adduct formation, MS, data processing, statistical analysis, and bioinformatics. Food lipidomics has successfully analyzed numerous plant- and animal-based foods containing diverse lipid molecules, thereby facilitating the identification and quantification of lipid species in these foods. In addition, this review provides several examples of MS-based lipidomics approaches used for determining lipid profiles in foods, especially TAG and phospholipid species, thereby highlighting the efficiency of this technique. Currently, in MS, a single injection enables the detection of more than a thousand compounds in the analyzed sample; however, an insufficient database limits the identification of the desired compounds in samples. Considerable efforts are currently being taken to produce a large database containing extensive information on MS-derived data in related fields. Thus, advanced MS analysis combined with diverse software tools may greatly enhance the detection range of lipid molecules in foods and the efficient observation of the changes in bioactive lipid species in foods.

## Figures and Tables

**Table 1:** Triacylglycerols and phospholipids found in plant-based foods.

Sample	Lipid Extraction	Lipidomics Approach	Main Finding	Reference
Triacylglycerols in Plant-Based Foods				
Soy milk	Folch and Bligh method	Ultra-performance liquid chromatography (UPLC)–Q-Exactive Orbitrap mass spectrometry (MS)	14 lipid molecules were selected as the markers for detecting milk fraud	[64]
Soybean oil	Bligh and Dyer method	Shotgun MS-based lipidomics	Identification and quantification of 93 TAG molecules, including 18:2–18:2–18:2 (LLL), 18:2–18:2–18:3 (LLLn), 18:1–18:2–18:2 (OLL), 16:0–18:2–18:2 (OLLn), 16:0–18:1–18:2 (POL), 18:1–18:1–18:2 (OOL), and 18:0–18:2–18:2 (SLL))	[65]
Hazelnut oil	Chloroform/methanol (2:1, *v*/*v*)	Chiral HPLC–atmospheric pressure chemical ionization (APCI)–MS coupled with two cellulose tris-(3,5-dimethylphenylcarbamate) columns in a series	Unsaturated fatty acids localized at sn-2 position of TAG structures in hazelnut oil	[66]
High-oleic and normal peanut oils	-	APCI-MS	Differences in TAG species between high-oleic and normal peanut oils (e.g., 18:1–18:1–18:1, 16:0–18:1–18:2, and 18:1–16:0–18:1)	[67]
Structured lipids	-	APCI-MS/MS	The level of 1,3-di-oleoyl-2-palmitoylglycerol was monitored in the synthesis of structured lipids	[68]
Phospholipids in plant-based foods				
Extra-virgin olive oil	n-Hexane and ethanol/water (80:20, *v*/*v*).	Exactive hybrid quadrupole–Orbitrap MS	Identification of 19 phospholipids	[69]
Almond	Bligh and Dyer method	Matrix-assisted laser desorption ionization–time-of-flight MS (MALDI-TOF/MS)	Identification of more than 60 phospholipid molecules Proposed that the ratio of m/z 833.6 to 835.6 and m/z 821.6 could be an efficient marker to distinguish almonds from different geographical origins	[70]
Olive (*Olea europaea* L.)	Bligh and Dyer method	High-performance liquid chromatography (HPLC)–electrospray ionization (ESI)-MS/MS	A total of 107 polar lipids belonging to 11 lipid classes, including phospholipids, glyceroglycolipids, glycosphingolipids, and betaine lipids in *O. europaea*	[71]
*Coffea canephora* L. beans	Methyl-*tert*-butyl ether (MTBE)	UPLC-MS	PE (34:2) is the predominant phospholipid species in *C. canephora* beans	[72]
Beans (soybean, red kidney bean, red bean, white kidney bean, chickpea, and black beans)	Chloroform/methanol (2:1, *v*/*v*)	UHPLC-Q-Exactive Orbitrap/MS	49 PC species were identified, 16:0_18:1_PC was the major PC species in chickpea	[73]
*Arabidopsis thaliana*	Butanol/methanol (1:1) with 10 mM ammonium formate	ESI/APCI-QqTOF- mass spectrometer	The alteration of lipid species in the model plant *A. thaliana* at several different developmental stages was monitored, and more than 200 lipid species were identified.	[74]

**Table 2:** Triacylglycerols and phospholipids found in animal-based foods.

Sample	Lipid Extraction	Lipidomics Approach	Main Finding	Reference
Triacylglycerols from Animal-Based Foods				
Pork	Dichloromethane/methanol (50:50, *v*/*v*)	UHPLC-QTOF	8:0_8:0_34:6 is the major TAG molecule in pork 32 TAG species were altered by changes in diet in pigs	[76]
Beef	-	Rapid evaporative ionization MS (REIMS)-based analysis	REIMS-based analysis showed a high accuracy in detecting meat frauds	[77]
Salmon muscle	Chloroform: methanol (2:1)	Shotgun-based lipidomics using ESI-MS/MS	Identification of 98 TAG molecules in salmon muscle tissue and their quantitative analysis using multiple neutral-loss scans	[32]
Bigeye tuna, bighead carp, and Atlantic salmon heads	Modified Folch method	Q Exactive MS system coupled with UHPLC	Identification of 87–146 TAG species in the tested marine sources	[38]
Mediterranean mussel (*Mytilus galloprovincialis*)	Bligh and Dyer method	ESI-MS/MS	Identification of 34 TAG molecules using product ion scanning mode	[78]
Anchovy and tuna oils	-	HPLC-APCI/MS	Identification and quantification of 23 TAG molecules Identification of DHA- and EPA-containing TAGs (e.g., 16:0_18:1_20:5, 18:1_22:6_22:6, and 16:0_18:0_22:6)	[79]
Phospholipids from animal-based foods				
Shrimp waste	Bligh and Dyer and Folch MTBE	GC-FID (HILIC)-QTrap-MS	Identification of 14 PCs, 14 PEs, 9 PIs, and 9 PSs	[84]
Salmon muscle	Folch method	Shotgun lipidomics using Triple Quadrupole Mass Spectrometer	Identification of 12 PCs, 14 Pes, 5 PSs, and 2 PIs Finding major species, including 16:0–22:6 PC, 18:1–20:5 PE, 18:2–20:4 PE, and 22:5–22:6 PS	[2]
Atlantic salmon king salmon rainbow trout	-	Quadrupole TOF-MS	Determination of predominant phospholipids, such as 18:0_20:5_PE 18:1_20:5_PE, 18:0_20:5_PE, 18:0_22:6_PE, and 18:0_22:6_ PI	[85]
Sea fish, freshwater fish, and shellfish	Bligh and Dyer method	HPLC-ESI-MS/ MS	Identification of 18 PCs, 24 PEs, 15 PSs, and 8 PIs	[86]
Crabs	Bligh and Dyer method	Hydrophilic interaction chromatography-MS (HILIC-MS)	Identification of 21 PCs, 9 PEs, 2 PSs, and 6 PIs, as well as EPA- and DHA-containing PLs, including 18:0_20:5_PC, 18:1_20:5_PE 16:0_22:6_PE, and 18:0_20:5_PI	[89]
Shrimp head, codfish roe, and squid gonad	Bligh and Dyer method	UPLC-Q-Exactive Orbitrap/MS	Identification of 310 phospholipid molecules with 34 different fatty acyl chain combinations	[87]
Sheep meat	100% isopropanol alcohol	UPLC-QOrbitrap HRMS	Finding a total of 90 lipids belonging to six lipid subclasses, such as sphingomyelin, ceramide, lysophosphatidylcholine, phosphatidylcholine, phosphatidylethanolamines, and triacylglycerol	[90]
Duck	Methanol: Chloroform (2:1)	ESI-MS/MS	Identification and quantification of a total of 118 phospholipid species in duck meat	[88]
*Chlorella vulgaris*	Ethanol dichloromethane:methanol (2:1, *v*/*v*)	HILIC–ESI–MS	More than 30 phospholipids were identified in the lipid extract of *C. vulgaris* Using ultrasonication in lipid extraction increased the yields of polar lipids from *C. vulgaris*	[91]

## Data Availability

Not applicable.
